# Metabolic engineering of *Escherichia coli* for the production of 5-aminolevulinic acid based on combined metabolic pathway modification and reporter-guided mutant selection (RGMS)

**DOI:** 10.1186/s13068-024-02530-4

**Published:** 2024-06-17

**Authors:** Yuting Yang, Yuhong Zou, Xi Chen, Haidong Sun, Xia Hua, Lee Johnston, Xiangfang Zeng, Shiyan Qiao, Changchuan Ye

**Affiliations:** 1 State Key Laboratory of Animal Nutrition and Feeding, Ministry of Agriculture Feed Industry Centre, China Agricultural University, Beijing, 100193 China; 2 Beijing Key Laboratory of Bio-Feed Additives, Beijing, 100193 China; 3 State Key Laboratory for Agro-Biotechnology, Ministry of Agriculture and Rural Affairs, Key Laboratory for Pest Monitoring and Green Management, Department of Plant Pathology, China Agricultural University, Beijing, 100193 China; 4 National Feed Engineering Technology Research Centre, Beijing, 100193 China; 5 Swine Nutrition and Production, West Central Research and Outreach Center, University of Minnesota, Morris, MN 56267 USA; 6 Department of Animal Science, College of Animal Science, Fujian Agriculture and Forestry University, Fuzhou, 350002 China

**Keywords:** *E. coli*, 5-Aminolevulinic acid, CRISPR/cas9, Metabolic engineering, Reporter-guided mutant selection, Promoter modification

## Abstract

**Background:**

5-Aminolevulinic acid (ALA) recently received much attention due to its potential application in many fields such as medicine, nutrition and agriculture. Metabolic engineering is an efficient strategy to improve microbial production of 5-ALA.

**Results:**

In this study, an ALA production strain of *Escherichia coli* was constructed by rational metabolic engineering and stepwise improvement. A metabolic strategy to produce ALA directly from glucose in this recombinant *E. coli* via both C4 and C5 pathways was applied herein. The expression of a modified *hemA*^*RS*^ gene and rational metabolic engineering by gene knockouts significantly improved ALA production from 765.9 to 2056.1 mg/L. Next, we tried to improve ALA production by RGMS-directed evolution of *eamA* gene. After RGMS, the ALA yield of strain A2-ASK reached 2471.3 mg/L in flask. Then, we aimed to improve the oxidation resistance of cells by overexpressing *sodB* and *katE* genes and ALA yield reached 2703.8 mg/L. A final attempt is to replace original promoter of *hemB* gene in genome with a weaker one to decrease its expression. After 24 h cultivation, a high ALA yield of 19.02 g/L was achieved by 108-ASK in a 5 L fermenter.

**Conclusions:**

These results suggested that an industrially competitive strain can be efficiently developed by metabolic engineering based on combined rational modification and optimization of gene expression.

**Supplementary Information:**

The online version contains supplementary material available at 10.1186/s13068-024-02530-4.

## Background

**Fig. 1:**
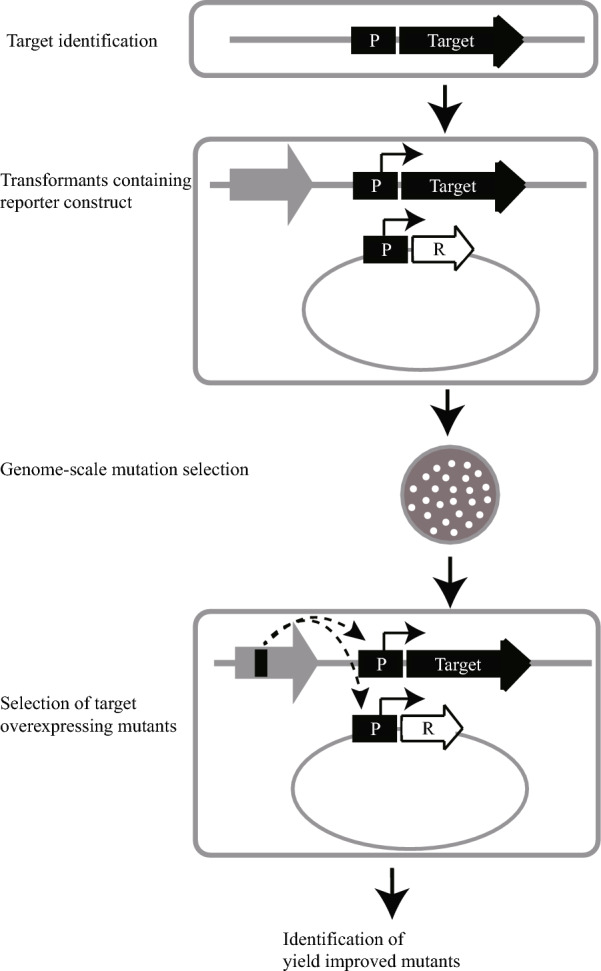
Schematic illustration of the principle of RGMS. RGMS means reporter-guided mutant selection

Synthetic biology plays a critical part in bio-based production of fuels, chemicals and materials from biomass. Biologists could construct cell factories by applying engineering principles and methods [1–3] to produce medicinally relevant compounds (strictosamide, taxadiene, etc.) [4, 5] or amino acids (L-valine, L-threonine, etc.) [6, 7] in a high titer. With the development of recombinant DNA technology and improved understanding of metabolism and regulation, metabolic engineering has emerged as an efficient strategy [8, 9]. Metabolic engineering is based on rational genetic modification of metabolic pathways. However, the outcomes of these rational modifications do not always agree with assumptions, as metabolite titer is a complex trait influenced by diverse factors [10]. As a strategy to improve the success rate of metabolic engineering, inverse metabolic engineering became a favored tool of biologists [11, 12]. The ration of it is to identify key gene targets conferring a desired phenotype, and then endow such a phenotype on another strain by directed genetic manipulation [13, 14]. The reporter-guided mutant selection (RGMS) method is a developed tool of inverse metabolic engineering (Fig. 1), which combines the merits of random mutagenesis and rational mutant selection [13]. The central rationale of RGMS is to convert signals of target expression into those of reporter expression and then use this signal conversion to facilitate mutant selection [10].

In this study, we reported the application of combined RGMS and metabolic engineering in the selection of 5-aminolevulinic acid (ALA) over-producing mutants from *E. coli*. ALA is a key intermediate involved in the biosynthesis of tetrapyrrole, which has attracted much attention for its potential applications in agriculture, cosmetics and cancer therapy. ALA is also used as selective herbicide and insecticide in agriculture due to its nontoxicity and biodegradability [15]. In living organisms, there are two major pathways described for ALA biosynthesis [15]. One is the C4 pathway, which occurs in mammals, birds, yeast, and purple non-sulfur photosynthetic bacteria. In this pathway, ALA is formed by condensing glycine and succinyl-CoA through catalyzation of 5-aminolevulinate synthase (ALAS) [16]. The second is C5 pathway, which is present in higher plants, algae and many bacteria, including *E. coli* [17]. In the C5 pathway, glutamate is the only substrate for biosynthesis of ALA.

In our earlier work, we developed an available metabolic strategy of ALA biosynthesis by over-expressing *hemA*, *hemL* and *eamA* genes. We found that the heterologous expression of *hemA*^*RS*^ could significantly improve ALA yield [18, 19]. In the research reported herein, we used a similar process for ALA biosynthesis in *E. coli* BW25113-T7. Combined rational metabolic engineering and RGMS methods were applied herein to develop an over-producing mutant of *E. coli* strain.

## Results

### Available metabolic strategy of ALA production in *E. coli* by overexpressing *hemA*,* hemL*, *eamA* and *hemA*^*RS*^

**Fig. 2:**
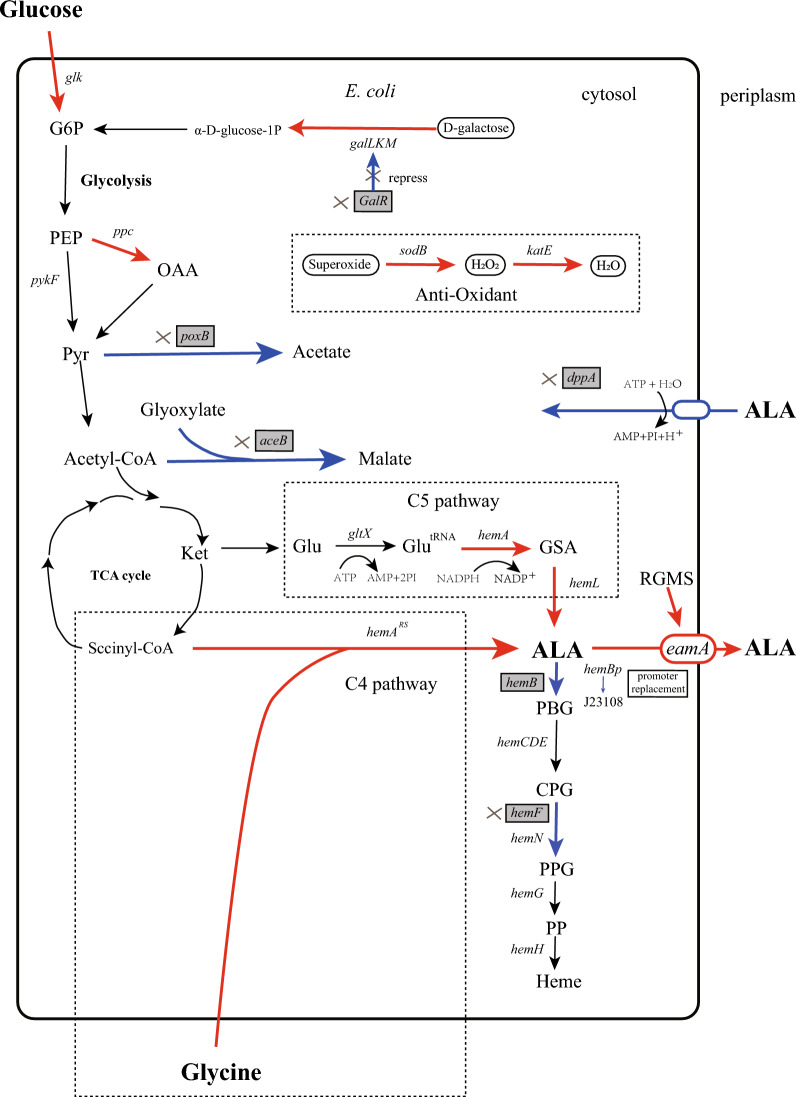
Biosynthetic pathways of ALA in *E. coli* and the strategies of metabolic engineering. The shaded boxes indicate the genes that were knocked out or inhibited. Red arrows indicate increased flux or activity by directly over-expressing the corresponding genes. Blue arrows indicate decreased flux or activity by knocking out the corresponding genes. Dotted boxes represent the corresponding metabolic pathways

ALA can be synthesized in *E. coli* via the C5 pathway from glutamate (Fig. 2). We first constructed an ALA-producing *E. coli* base strain (*E. coli* BWT7-RSA) from BW25113-T7 by overexpressing *hemA*, *hemL*, *eamA* and *hemA*^*RS*^ separately using two multi-copy plasmids (pET28b-LAA and pACYCD-RSA). The amino acid sequence of *hemA*^*RS*^ was modified for better expression in *E. coli*. The *E. coli* BW25113-T7 strain applied here was constructed in our earlier work which achieved high efficiency of protein expression through T7 expression system [19]. After 24 h of induced fermentation in flask, an ALA titer (765.94 mg/L) was determined (Table 1).

**Table 1:** ALA production in recombinant *E. coli* expressing various related genes

Strains	Character	Expressed genes	ALA concentration (mg/L)
BWT7-RSA	*E. coli* BW25113-T7	*hemA, hemL, eamA*	765.94 ± 25.16
A2-RSA	*E. coli* A2 (*E. coli* BW25113-T7 Δ*aceB*, Δ*dppA*, Δ*hemF*, Δ*galR* int *ppc* with J23119, Δ*poxB* int *glk* with J23119)	*hemA, hemL, eamA, hemARS*	2056.11 ± 59.48
BWT7-LAA	*E. coli* BW25113-T7	*hemA, hemL, eamA*	718.05 ± 9.17
BWT7-LAA(C)	*E. coli* BW25113-T7	*hemA, hemL, eamA(C)*	804.17 ± 13.21
BWT7-LAA(C1)	*E. coli* BW25113-T7	*hemA, hemL, eamA(C1)*	846.68 ± 12.16
A2-RSA-C1	*E. coli* A2	*hemA, hemL, eamA(C1), hemARS*	2471.33 ± 60.31
A2-ASK	*E. coli* A2	*hemA, hemL, eamA(C1), hemARS, katE, sodB*	2703.75 ± 93.59
108-ASK	*E. coli* 108 (*E. coli* A2 Δ*PhemB*, Int::J23108)	*hemA, hemL, eamA(C1), hemARS, katE, sodB*	3267.09 ± 105.48
111-ASK	*E. coli* 111 (*E. coli* A2 Δ*PhemB*, Int::J23111)	*hemA, hemL, eamA(C1), hemARS, katE, sodB*	3434.19 ± 137.88
116-ASK	*E. coli* 116 (*E. coli* A2 Δ*PhemB*, Int::J23116)	*hemA, hemL, eamA(C1), hemARS, katE, sodB*	2245.29 ± 123.34

A 1% (v/v) inoculum from an overnight culture for 12 h was used. IPTG was added in 4 h when OD_600_ reached 0.7. Samples were taken and measured until 24 h. 10 g/L glucose was added initially as sole carbon source. Glycine (2 g/L) as substrate for C4 pathway was added as indicated. The results are the average of three individual experiments

### Enhanced production of ALA by metabolic pathway modification

The biosynthetic pathways of TCA cycle in *E. coli*, the regulations involved, and the strategies for constructing ALA production strain are shown in Fig. 2. The *E. coli* BWT7-RSA strain was rationally engineered to produce a higher titer of ALA by the following targeted genetic modifications.

Based on the published metabolic and regulatory information, we knocked out several genes responsible for the competing pathways (*aceB*, *dppA*, *hemF*, *galR* and *poxB*). Then, two genes (*ppc* and *glk*) encoding those enzymes directly involved in glycolysis and pyruvate metabolism were amplified and inserted by chromosomal replacement (Fig. 2). After targeted genetic modifications, the A2 strain (*E. coli* BW25113-T7 Δ*aceB*, Δ*dppA*, Δ*hemF*, Δ*galR* int *ppc* with J23119, Δ*poxB* int *glk* with J23119) was constructed. Growth of *E. coli* A2 in different medium was examined to assess whether CRISPR/Cas9-mediated gene knock-out affected the metabolic characteristics of the bacteria. There were no differences in growth rate among two strains (BW25113-T7 and A2) in LB and CAYE medium (Fig. S1). These results indicated that metabolic pathway modification based on known metabolic and regulatory information does not impact growth characteristics of *E. coli.*

To examine the performance of the A2 strain, batch cultures of this recombinant strain were carried out in CAYE medium containing 10 g/L glucose. The final ALA concentrations obtained with this strain were 2,056.1 mg/L (Table 1). After pathway was modified, the ALA concentration obtained with the A2-RSA (harbored pET28b-LAA and pACYCD-RSA) was 2.27-fold higher than that obtained with the corresponding recombinant BWT7-RSA strain. The ALA yield achieved with A2-RSA (pET28b-LAA, pACYCD-RSA) was as high as 0.206 g of ALA per gram of glucose.

### Modified single-reporter RGMS

**Fig. 3:**
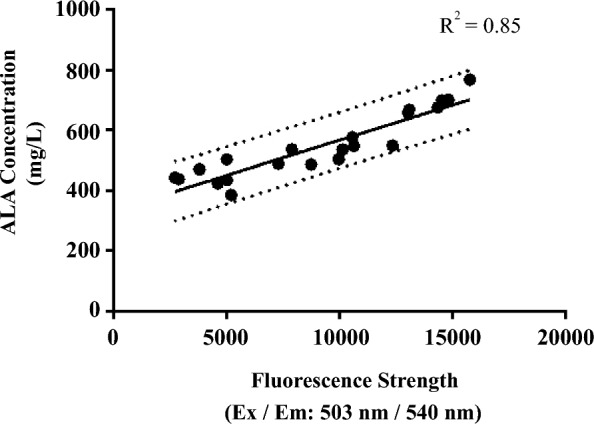
Sensitivity of BioSensor *PhemL*. The dotted line shows the 95% prediction bands of the best-fit line. The *P* value of slope deviation from zero is less than 0.05. *E. coli* BW25113-T7 containing pSC-LA-eamA and pUC57-sYFP-pHemL was used

Previous studies have indicated that *hemL* has the potential to integrate multiple signals to regulate ALA biosynthesis. This critical gene encodes HemL, which catalyzes the transfer of the amino group of glutamate-1-semialdehyde (GSA) to form ALA [20] and suggesting metabolic channeling of the highly reactive pathway intermediate GSA [21]. The activity of this key enzyme for ALA biosynthesis is commonly inhibited by heme as ALA accumulation was regulated by feedback inhibition of heme [22–24]. Based on above metabolic information, we supposed the expression of *hemL* was regulated both by ALA and heme. To verify our hypothesis, we conduct an experiment to test the relation between *hemL* expression and ALA concentration by fluorescent reporter gene system. In this study, we chose sYFP (Yellow Fluorescent Protein) as the reporter for RGMS. The promoter of *hemL* (*PhemL*) was used to construct the reporter plasmid (pUC57-sYFP-pHemL). A linear correlation was found between the fluorescent signal and ALA concentration (Fig. 3). This result indicated that *PhemL* could a potential indicator of ALA over-production in our fermentation environment.

In our previous study, we discovered that overexpression of ALA exporter (such as *rhtA* and *eamA*) would increase the accumulation of ALA. As well, our results revealed that a higher rate of ALA export will increase ALA concentration in supernatant due to greater expression of exporter gene [18, 19]. Hence, we choose *eamA* gene as the target for RGMS, and its CDS was used to construct the mutation plasmid (pSC-LA-eamA).

**Fig. 4:**
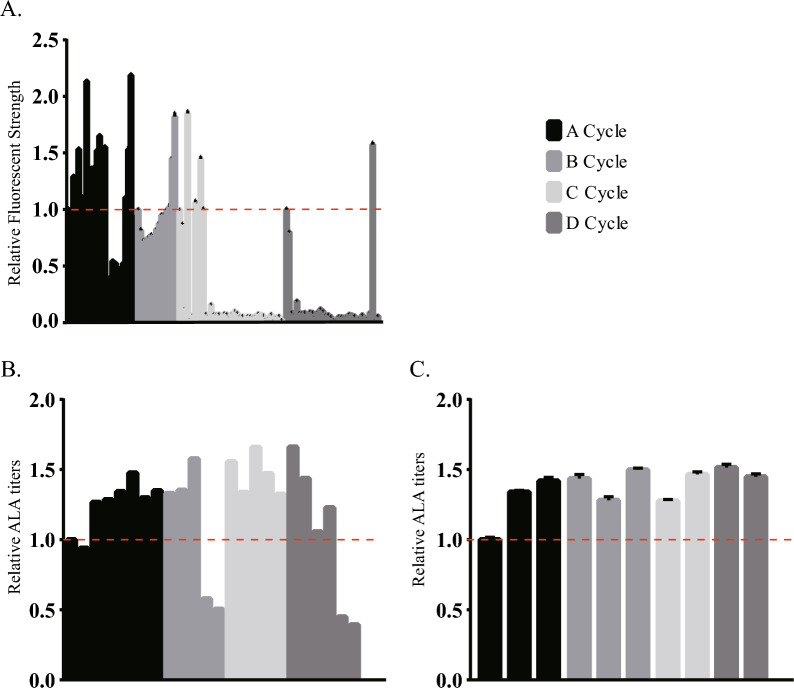
Results of RGMS. **A** Relative Fluorescent Strength of RGMS mutants. (*Y* axis: Relative Fluorescent Strength). **B** Approximately 6 ~ 10 mutants which showed strong fluorescence were selected to measure ALA production without replications. (*Y* axis: relative ALA titers). **C** 2 ~ 3 mutants which have high titers of ALA production were isolated and remeasured ALA accumulation with three replications. *Y* axis: relative ALA titers. The control was set as strains which harbored pSC-LA-eamA

In our modified single-reporter RGMS design, our object to develop a better mutant of *eamA* gene with a more efficient rate of ALA export. Thereafter, the mutation plasmid pSC-LA-eamA which contained the original *eamA* gene was subjected to RGMS as the starting plasmid. The schematic diagram of modified single-reporter RGMS process we design in this study was shown in Fig. S2. A total of four cycles were conducted in this experiment, named as A, B, C and D cycle, respectively. The results of RGMS were shown in Fig. 4.

Figure 4A shows the results of high-throughput screening of reporter genes. It can be observed that the efficiency of fluorescence screening decreases with the increase of RGMS cycles. It is difficult to screen mutants with enhanced fluorescence signals in D cycle. Figure 4B, C shows the results of twice ALA yield screenings. Similar to the results of fluorescence screening, the optimal efficiency of mutation screening effect was obtained in A cycle and the ALA yield increase could reach nearly 40%. However, the screening efficiency decreased dramatically with the increase of RGMS cycles. In D cycle, the ALA yield was only increased by 4% compared with control group. These results indicated that directed evolution of *eamA* was close to saturation after four cycles of RGMS without sequencing. Thus, we decided to terminate the cycles and select the optimal mutant gene, which was named *eamA(C)*.

### CDS repairing of *eamA(C)*

**Fig. 5:**
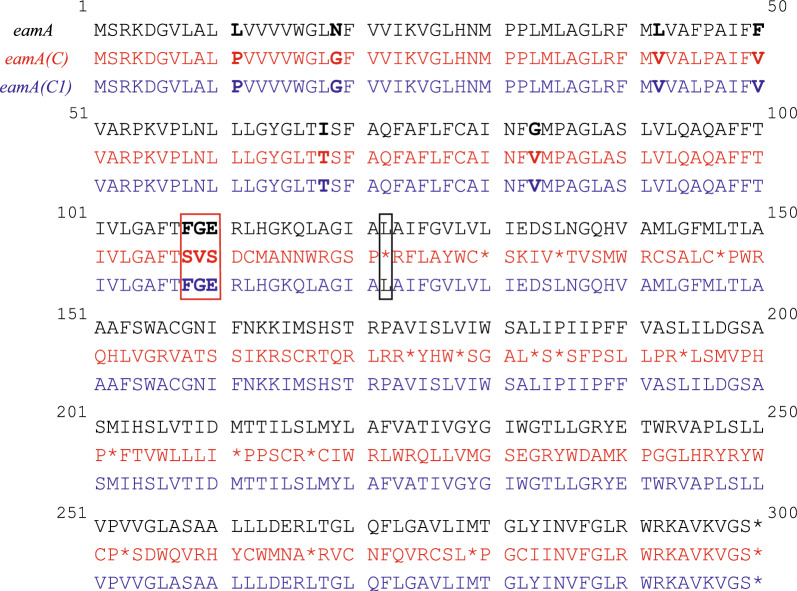
CDS translation of original *eamA* and its mutants. Positions in bold type indicate the mutations of amino acid. The red box indicates the position of frameshift mutation. The black box indicates the position of the stop codon which occurs earlier than the original *eamA*

We sequenced CDS of *eamA(C)* to find out why the screening efficiency decreased so dramatically. We found that *eamA(C)* had a deletion mutation in the base position 322 bp away from the start codon compared with *eamA*. This deletion mutation resulted in a lack of nearly 180 amino acids in the coding protein (shown in Fig. 5).

However, our result showed that this mutant *eamA(C)* still led to a good ALA titer. We supposed that 108 amino acids encoded by the preceding 321 bp (before deletion mutation) were conservative and have critical functions in ALA efflux. Besides, a total of 12 amino acids are mutated in CDS before the deletion mutation. These mutations may lead to the increase of ALA accumulation. Thus, we speculated the ALA efflux capacity of *eamA(C)* could be enhanced when the deletion mutation was repaired and further increased ALA production. Based on the above analysis, original *eamA* gene was used as template to repair the deletion mutation and subsequent CDS of *eamA(C)*. Thereafter, *eamA(C1)* was obtained (shown in Fig. 5).

**Fig. 6:**
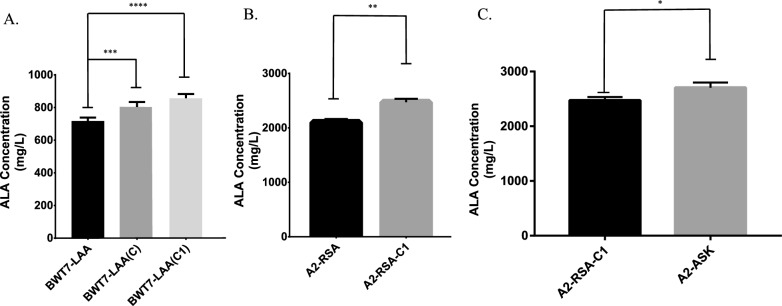
ALA titers of mutant strains. Adjusted *P* values were calculated using Dunnett’s multiple comparisons test (*P < 0.05, **P < 0.01, ***P < 0.001, ****P < 0.0001). Induced fermentation lasted for 24 h

We examined the ALA production of these two mutants, *eam(C)* and *eam(C1*), in BW25113-T7 (Table 1 and Fig. 6A). These three strains, BWT7-LAA, BWT7-LAA(C) and BWT7-LAA(C1), all overexpressed *hemL* and *hemA* genes. BWT7-LAA overexpressed *eamA*, BWT7-LAA(C) overexpressed *eamA(C)* and BWT7-LAA(Cc) overexpressed *eamA(C1),* respectively (Table 2). BWT7-LAA(C1) accumulated 17.9% more ALA than BWT7-LAA, which indicated that *eamA(C1)* has a better excretion efficiency of ALA than *eamA* (*P* < 0.001). Then, we replaced pET28b-LAA with pET28b-LA-eamA(C1) in *E. coli* A2-RSA to obtain *E. coli* A2-RSA-C1. ALA accumulation in recombinant strain A2-RSA-C1 reached 2,471.3 mg/L (Table 1 and Fig. 6B**)**. These results indicated that *eamA(C1)* obtained by RGMS and CDS repairing had a greater capacity for ALA excretion and further improved accumulation of ALA in CAYE medium.

**Table 2:** Batch fermentation of ALA in *E. coli* A2-ASK, 108-ASK and 111-ASK in a 5 L fermenter

Strain	Expressed genes	Cell biomass (OD600)	ALA concentration (g/L)
*E. coli* A2	*hemA, hemL, eamA(C1), hemARS, katE, sodB*	32.76 ± 0.44	13.62 ± 0.62
*E. coli* 108 (*E. coli* A2 Δ*PhemB*, Int::J23108)	*hemA, hemL, eamA(C1), hemARS, katE, sodB*	32.20 ± 0.53	19.02 ± 0.38
*E. coli* 111 (*E. coli* A2 Δ*PhemB*, Int::J23111)	*hemA, hemL, eamA(C1), hemARS, katE, sodB*	31.90 ± 0.78	10.73 ± 0.36

Strain	ALA production (g)	Glucose consumption (g)	ALA production rate (g/g)
*E. coli* A2	34.50 ± 1.55	222.24 ± 2.00	0.155 ± 0.007
*E. coli* 108 (*E. coli* A2 Δ*PhemB*, Int::J23108)	49.00 ± 0.89	235.70 ± 0.71	0.208 ± 0.003
*E. coli* 111 (*E. coli* A2 Δ*PhemB*, Int::J23111)	26.76 ± 0.98	196.95 ± 2.75	0.136 ± 0.007

A 10% (v/v) inoculum from seed cultures was used. 20 g/L glucose and 2 g/L glycine were added initially. 0.1 mM IPTG was added as indicated. During the fermentation, the pH was controlled optimally at 6.0

### Synergetic effect of *sodB* and *katE* on ALA production

An earlier study demonstrated that high concentration of ALA caused severe cell damage and morphological change of *E. coli* via generating reactive oxygen species (ROS) [25]. The existence of PoxB may decrease oxidative stress under aerobic conditions [26]. As we have knocked out *poxB* gene in A2 strain for a better flux of pyruvic acid, A2 strain may suffer severe oxidative stress. Thus, we speculated that an enhanced antioxidant defense system by overexpressing key genes *sodB* and *katE* could compensate and improve overall antioxidant capacity of cells and tolerance of ALA in high-density fermentation. We believed this metabolic strategy would lead to an increase of its production level. In this study, we constructed a new plasmid pACYCD-ASK, which overexpressed *hemA*^*RS*^, *katE* and *sodB* synergistically. This plasmid and pET28b-LA-eamA(C) were both introduced to *E. coli* A2 strain to obtain A2-ASK. Notably, co-expression of *katE* and *sodB* in an ALA synthase expressing strain (A2-ASK) enhanced final ALA titer 9.4% (2703.8 mg/L) in flask fermentation (*P* < 0.05), compared with A2-RSA-C1 (Table 1 and Fig. 6C). This result revealed that an enhanced antioxidant defense system would increase the accumulation of compound that may cause oxidative stress such as ALA.

### Suppression of *hemB* gene

**Fig. 7:**
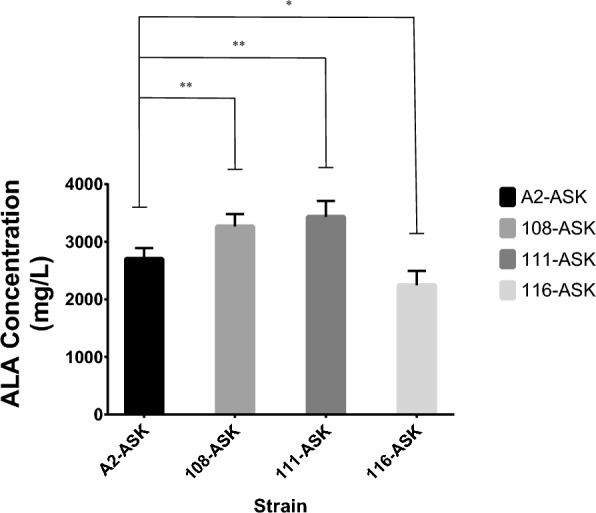
ALA titers of 108-ASK, 111-ASK and 116-ASK strains. Adjusted *P* values were calculated using Dunnett’s multiple comparisons test (*P < 0.05, **P < 0.01, ***P < 0.001, ****P < 0.0001). A2-ASK was set as control group. Induced fermentation lasted for 24 h

**Fig. 8:**
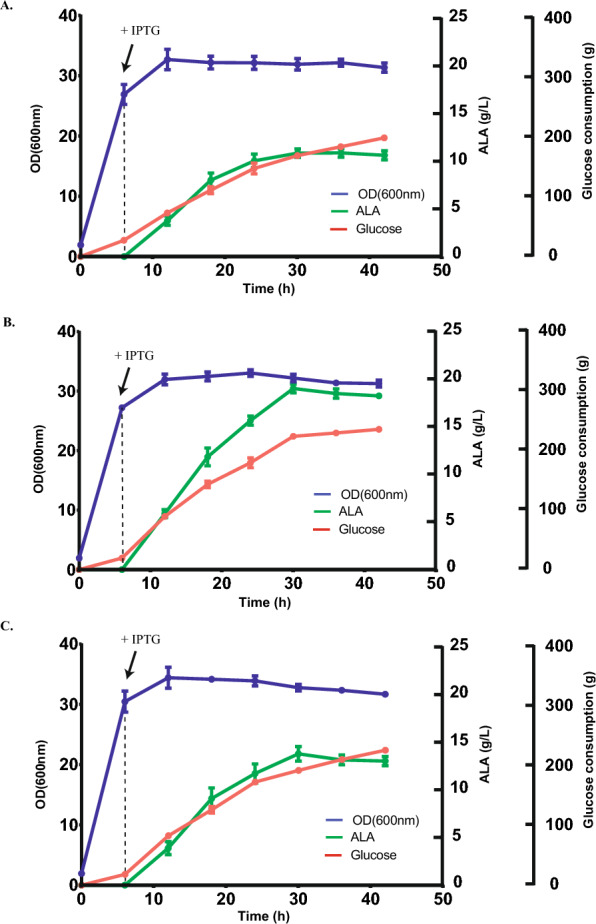
Batch fermentation of ALA in *E. coli* A2-ASK (**A**), 108-ASK (**B**) and 111-ASK (**C**) in a 5 L fermenter. A 10% (v/v) inoculum from seed cultures was used. 20 g/L glucose and 2 g/L glycine were added initially. 0.1 mM IPTG was added as indicated. During the fermentation, the pH was controlled optimally at 6.0

Releasing the feedback inhibition of ALA synthesis by heme is expected to further improve ALA production [23, 24]. The inhibition of *hemB* gene would mightily repress the biosynthesis of porphobilinogen (PBG) resulting in reduced endogenous loss of ALA [27–29]. In this study, we managed to replace *PhemB* with constitutive promoter (J23108, J23111 or J23116) via CRISPR/Cas mediated gene editing (Fig. 2). The expression levels of these constitutive promoter (Table S1) were acquired from iGEM, which is available at http://parts.igem.org/Promoters. *PhemB* in genome of *E. coli* A2 was replaced with J23108, J23111 or J23116. The mutants were named as *E. coli* 108, *E. coli* 111 or *E. coli* 116, respectively. We also found no differences in growth rate among these strains (BW25113-T7, A2, 108, 111 and 116) in LB and CAYE medium (Fig. S1). Subsequently, pET28b-LA-eamA(C1) and pACYCD-ASK were both introduced to *E. coli* 108, 111 or 116 strain to obtain *E. coli* 108-ASK, 111-ASK or 116-ASK. Then ALA accumulations of these strains were examined in flask fermentation (Table 1 and Fig. 7). Out of our expectation, 116-ASK showed the lowest ALA accumulations among these mutants although expression levels of J23116 was weaker than J23111 and J23108. The other two mutants, 108-ASK and 111-ASK, both demonstrated better production of ALA (*P* < 0.01). The strains 108-ASK and 111-ASK accumulated 20.8% and 27.0% more ALA, respectively, than the strain A2-ASK. To further improve ALA production, these three strains (A2-ASK, 108-ASK and 111-ASK) were fermented in 5 L fermenter and glucose was added with an initial concentration of 20 g/L. After 42 h cultivation, a high titer of ALA (19.02 g/L) with a yield of 0.208 g ALA per g glucose was achieved by 108-ASK (Table 2 and Fig. 8).

To confirm our assumption, we compared expression levels between these two promoters (*PhemB* and J23108) by fluorescent reporter gene system. The expression level of J23108 was weaker than *PhemB* both in LB and CAYE medium (shown in Fig. S3). These results revealed that replacing the original promoter with a weaker constitutive promoter (J23108) to inhibit *hemB* gene would improve ALA accumulation.

## Discussion

Traditionally, recombinant engineering strains have been developed by random mutagenesis and selection. Although various large-scale analytical techniques such as transcriptome and proteome analysis are now available, it is difficult to apply these techniques on the randomly mutated industrial strains for further strain improvement because of unknown mutations in their genome [6]. Thus, the aim of this study was to construct a genetically well-defined engineered *E. coli* strain based on known regulatory information, metabolic pathway modification and directed protein optimization by RGMS. Although *E. coli* has complex regulatory mechanisms for ALA biosynthesis, many known regulatory mechanisms could be engineered toward enhanced ALA production. After the systematic metabolic engineering, recombinant protein expression, random mutation and selection of *E. coli*, we were able to achieve a high yield of 0.208 g of ALA per gram of glucose. More importantly, the engineered strain developed in this study can be further improved because all of the modifications are clearly defined.

At first, we constructed the ALA-producing *E. coli* base strain with key genes (*eamA*, *hemA*, *hemL* and *hemA*^*RS*^) overexpression base on our previous research [18, 19]. EamA (encoded by *eamA* gene) is an O-acetylserine/cysteine exporter which is capable of translocating dipeptides and amino acid analogs from the cytosol to the periplasm. It was reported that overexpression of *eamA* gene would enhance ALA accumulation in the supernatant [18]. Based on our previous study, synergistically produced ALA via the C4 pathway could further increase accumulation of ALA [18]. ALA production was increased from 1601.7 to 2099.7 mg/L by heterologous expression of *hemA*^*RS*^, a key enzyme from *Rhodobacter sphaeroides*, with a 31.1% increase in ALA accumulation [18].

Then this base strain *E. coli* BWT7-RSA was improved stepwise by metabolic engineering based on metabolic and regulatory information available in the literature (Fig. 2). In *E. coli*, ALA biosynthesis is tightly regulated by feedback inhibition of heme, the end product of the C5 pathway [23, 24, 30]. The inhibition of *hemF* gene was expected to reduce the flux of heme and improve ALA production. The dipeptide binding protein—*dppA*, actively import ALA through an interaction with the dipeptide inner membrane ATP-binding cassette transporter, *DppBCDF*, in *E. coli* [31]. Our previous work has proved that the knock-out of *hemF* and *dppA* would increase ALA production significantly [18].

Malate synthase A (encoded by *aceB* gene) catalyzes the condensation of acetyl-CoA with glyoxylate to produce (S)-malate and coenzyme A (CoA) [32]. Knockout of *aceB* reduces the production of (S)-malate from glyoxylate cycle but also declines CoA. There are many reactions known to produce CoA [33]. However, there are only two isozymes (encoded by *aceB* and *glcB*) known to produce (S)-malate [34, 35]. In this case, we hypothesized that knock-out of *aceB* would obtain a better utilization of glucose for TCA cycle and have a positive effect in ALA biosynthesis. Pyruvate oxidase (encoded by *poxB* gene) catalyzes the oxidative decarboxylation of pyruvate to form acetate and CO_2_ [34]. Under aerobic conditions, the pyruvate oxidase route is more critical for pyruvate metabolism than the pyruvate dehydrogenase route [36]. We hypothesized that knocking out *poxB* gene would reduce the flux of acetate and decrease endogenous loss of pyruvic acid in aerobic conditions, which may lead to an increased accumulation of ALA.

GalR (encoded by *galR* gene) is a DNA-binding transcription factor that represses the *galTKM* operon in the absence of D-galactose [37–40]. The enzymes encode by *galTKM* genes could endogenously catalyze interconversion reactions in galactose catabolism to synthesize α-D-glucose-6P (Fig. S4). Thus, we hypothesized that silence of *galR* gene in the absence of D-galactose can increase flux of α-D-glucose-6P, thereby positively affecting ALA biosynthesis (Fig. 2).

We managed to over-express *glk* and *ppc* genes by CRISPR/Cas9 mediated gene knock-in. Glucokinase (encoded by *glk* gene) phosphorylates glucose. Growth on other carbon sources does not appear to affect *glk* expression expect glucose. However, in this study we provide glucose as the main carbon source, which will lead to a reduced expression of *glk* by 50% [41]. In order to improve expression of *glk*, we try to insert *glk* gene promoted by J23119 promoter (the strongest promoter from constitutive promoter family) in genome. Phosphoenolpyruvate carboxylase (Ppc, encoded by *ppc* gene) replenishes oxaloacetate in TCA cycle. Researchers have reported that overexpression of *ppc* gene could improve the growth of *E. coli* on glucose carbon sources [42], increases production of succinate [43] and reduces acetate excretion [44]. In this study, we also managed to insert *ppc* gene (promoted by J23119 promoter) in genome to better utilize glucose. After the manipulation of all these known and rationally selected genes, a mutant strain named *E. coli* A2 was obtained. As expected, these designed metabolic pathway modifications of *E. coli* genome enhanced ALA production by 168.4% (Table 1).

Further improvement of the ALA-producing strain was achieved by RGMS as no further engineering targets could be easily identified. RGMS was first used to improve lovastatin production in a filamentous fungus [13]. The classic RGMS method will make random mutagenesis in the genome which may be a potential drawback of this method. It would introduce both beneficial and harmful mutations into the genome. Besides, the mutation site would be untraceable via a completely random mutation. In this study, we constructed a modified single-reporter RGMS method on *E. coli*. The yellow fluorescent protein (sYFP) was selected as the reporter gene and the promoter *hemL* gene was selected as BioSensor. Only the targeted gene (*eamA*) was mutated by mutagenic PCR. This optimized RGMS method ensured the traceability of the mutation site and the ALA production of this recombinant strain A2-RSA-C1 could reach 2.47 g/L.

In a previous study, it was suggested that ALA inhibits cell growth through generating ROS [25]. Researchers found that excessive accumulation of porphyrins (produced from ALA metabolism) would cause serious oxidative damage to bacteria as porphyrins have strong photosensitive activities. Bacteria have developed a variety of defense mechanisms against oxidative stress to protect cells from the damage caused by ROS [45]. Superoxide dismutase (SOD, encoded by *sodA*, *sodB*, and *sodC*) could reduce superoxide anion radical to produce H_2_O_2_. And catalase (CAT, encoded by *katG* and *katE*) could further degrade H_2_O_2_ to water and oxygen [46]. With the knowledge of toxicity mechanism of ALA, we managed to implement effective measures to overcome its toxicity. Overexpression of *sodB* and *katE* genes in *E. coli* contributed a 9.4% (ca. 2.70 g/L) increase in ALA titer and prepared the host for high‐level ALA production, which demonstrated the importance of tolerance engineering in high‐level bio-production. We believed that the antioxidant defense system was a potential target for building a robust microbial platform.

HemB (encoded by *hemB* gene) catalyzes the synthesis of PBG from ALA and controls the overall metabolic flux downstream. Therefore, *hemB* gene plays a major part in controlling ALA biosynthesis [27–29]. However, knock-out of *hemB* gene would lead to extremely low PBG deaminase activity [47] and respiration-deficient [48] as *hemB* mutants cannot accumulate porphyrins [49]. Previous studied showed that decrease the expression of the *hemB* gene in varied degrees by expressing asRNAs (antisense RNAs) or CRISPRi would attenuate the synthesis of PBG and thus elevate the accumulation of ALA in *E. coli*. Eventually, the accumulation of ALA in *hemB*-weakened strains varied from 90.2% to 493.1% compared with original strain [50, 51]. Those works provide evidence for fine-tuning the heme biosynthesis pathway to enhance ALA accumulation. As *hemB* mutants lack ability of porphyrins accumulation [49] and are respiration-deficient [48], we managed to decrease expression of *hemB* by promoter replacement in the genome. The constitutive promoters (J23108, J23111, or J23116) were individually inserted into the genome of *E. coli* A2 to replace *PhemB* (the original promoter of *hemB* gene). Thereby we obtained the mutant strains *E. coli* 108, 111, or 116. Both 108-ASK and 111-ASK showed increased ALA production compared to control (A2-ASK). Beyond our expectation, mutant strain *E. coli* 116 showed the lowest ALA production among these strains although J23116 was the weakest promoter. We hypothesize that the over-weaken of *hemB* gene may cause a negative effect on heme metabolism and lead to a decreased ALA accumulation in mutant strain 116-ASK.

To produce ALA in these recombinant *E. coli* strains more efficiently, we optimized the cultivation condition and culture medium in batch fermentation (optimization process was omitted). After optimization, the ALA production was greatly enhanced in our modified batch fermentation (see methods and materials section). Cultivation of A2-ASK in modified batch fermentation (10.73 g/L) accumulated much more ALA than that in flask fermentation (2.70 g/L). ALA accumulation in the strain 108-ASK rose from 3.27 to 19.02 g/L, which showed the highest ALA titer among these strains (Table 2 and Fig. 8). After 42 h cultivation in our modified batch fermentation, a high titer of ALA (19.02 g/L) with a yield of 0.208 g ALA per g glucose was achieved (Fig. 8). A confirmatory experiment to test strength of promoters via reporter gene proved that J23108 was weaker than *PhemB*. These results revealed that a weaker promoter replacement of *hemB* gene would enhance ALA accumulation.

The conversion of best strain we constructed in this study reached 0.208 g ALA per g glucose. We believe that there remains potential for further enhancing ALA yield, encompassing strategies such as "rational design of key enzymes" and "systematic modification of metabolic pathways". As our work continues, we are actively optimizing the biosynthesis of ALA to create highly competitive engineered strains. Additionally, we are confident that further optimizing the fermentation process will elevate ALA production to even greater heights.

## Conclusion

In summary, rational metabolic engineering based on known metabolic and regulatory information, RGMS gene optimization, tolerance engineering and promoter replacement of a critical gene allowed development of an *E. coli* strain capable of efficiently producing ALA. This optimized strain achieved a high ALA production level of 19.02 g/L in a 5 L fermenter, demonstrating a productivity of 0 0.208 g ALA per gram of glucose. By further optimizing the fermentation process, we anticipate achieving even higher levels of ALA production. Moreover, the strategies outlined here have the potential to be widely applied in the development of strains for the efficient production of various other metabolites.

## Materials and methods

### Bacterial strains and plasmids

The bacterial strains and plasmids used in this study are listed in Table 3. *Escherichia coli* DH5a was used for general cloning. *E. coli* BW25113-T7 was used as the starting strain. Plasmids pET28b-LAA and pACYCD-RS-hemA were constructed in our previous study [18, 19] and were stored in our lab.

**Table 3:** Strains and plasmids employed in this study

Strain	Relevant characteristic(s)	Source or reference
*E. coli* DH5α	*F-,φ80dlacZ ΔM15,Δ(lacZYA -argF)U169, deoR, recA1, endA1,hsdR17 (rK-, mK* +*), phoA, supE44, λ-, thi-1, gyrA96, relA1*	TransGen, Beijing
*E. coli* BW25113-T7	*F-, DE(araD-araB)567, lacZ4787(del)::rrnB-3, LAM-, rph-1, DE(rhaD-rhaB)568, hsdR514, int:: (lacI:: PlacUV5:: T7 gene) ΔybhC*	Laboratory stock
*E. coli* A2	*E. coli* BW25113-T7 Δ*aceB*, Δ*dppA*, Δ*hemF*, Δ*galR* int *ppc* with J23119, Δ*poxB* int *glk* with J23119)	This study
*E. coli* 108	*E. coli* A2 Δ*PhemB*, Int::J23108	This study
*E. coli* 111	*E. coli* A2 Δ*PhemB*, Int::J23111	This study
*E. coli* 116	*E. coli* A2 Δ*PhemB*, Int::J23116	This study
*E. coli* BWT7-RSA	*E. coli* BW25113-T7 harboring pET28b-LAA and pACYCD-hemA-RS	This study
*E. coli* A2-RSA	*E. coli* A2 harboring pET28b-LAA and pACYCD-hemA-RS	This study
*E. coli* BWT7-LAA	*E. coli* BW25113-T7 harboring pET28b-LAA	This study
*E. coli* BWT7-LAA(C)	*E. coli* BW25113-T7 harboring pET28b-LA-eamA(C)	This study
*E. coli* BWT8-LAA(C1)	*E. coli* BW25113-T7 harboring pET28b-LA-eamA(C1)	This study
*E. coli* A2-RSA-C1	*E. coli* A2 harboring pET28b-LA-eamA(C1) and pACYCD-hemA-RS	This study
*E. coli* A2-ASK	*E. coli* A2 harboring pET28b-LA-eamA(C1) and pACYCD-ASK	This study
*E. coli* 108-ASK	*E. coli* 108 harboring pET28b-LA-eamA(C1) and pACYCD-ASK	This study
*E. coli* 111-ASK	*E. coli* 111 harboring pET28b-LA-eamA(C1) and pACYCD-ASK	This study
*E. coli* 116-ASK	*E. coli* 116 harboring pET28b-LA-eamA(C1) and pACYCD-ASK	This study
Plasmids	Relevant characteristic(s)	Source or reference
pUC57	Cloning vector, AmpR, ColE1/pMB1/pBR322/pUC ori	Laboratory stock
pACYCD	Cloning vector, CmR, p15a ori	Laboratory stock
pET-28b	Cloning vector, f1 ori; KanR	Laboratory stock
pUC57-sYFP	pUC57 with sYFP gene and J23107 promoter	Laboratory stock
pUC-sYFP-pHemL	pUC57 with sYFP gene and *PhemL* (promoter of *hemL* gene)	This study
pET28b-LAA	f1 ori; KanR; *hemA*; *hemL*; *eamA*; *LacI* gene and T7-LacI promoter	Laboratory stock
pSC-LA-eamA	temperature sensitive oriR101; KanR; *hemA*; *hemL*; *eamA*; *LacI* gene and T7-LacI promoter	This study
pET28b-LA-eamA(C)	f1 ori; KanR; *hemA*; *hemL*; *eamA(C)*; *LacI* gene and T7-LacI promoter	This study
pET28b-LA-eamA(C1)	f1 ori; KanR; *hemA*; *hemL*; *eamA(C1)*; *LacI* gene and T7-LacI promoter	This study
pCas	plasmid for CRISPR (temperature sensitive oriR101; KanR; the λ-Red operon under the control of arabinose-inducible promoter; *S. pyogenes*-derived cas9; sgRNA guided to ori-p15a under the control of lac operator)	Laboratory stock
pTarget-gene	plasmid for CRISPR (p15a ori; CmR; sgRNA guided to targeted gene, such as *hemF*, *aceB*, *poxB* gene and so on)	This study
pACYCD-gene	pACYCD containing targeted gene (such as *hemF*, *aceB*, *poxB* gene and so on)	This study
pACYCD-Donor DNA	pACYCD containing Donor DNA for targeted gene (such as *hemF*, *aceB*, *poxB* gene and so on)	This study
pACYCD-RS-hemA	pACYCD containing *hemA* from *R. sphaeroides* (modified) and T7-LacI promoter	This study
pACYCD-ASK	pACYCD containing *hemA* from *R. sphaeroides* (modified) and T7-LacI promoter; *sodB*, *katE* and J23107 promoter	This study

### Culture conditions

LB medium (10 g/L tryptone, 5 g/L yeast extract and 10 g/L NaCl, pH 7.2) was used in all DNA manipulations. During cultivation and fermentation, the citric acid medium (CAYE) was used that contains 1.86 g/L citric acid, 9 g/L KH_2_SO_4_, 6 g/L (NH_4_)_2_HPO_4_, 0.6 mg/L MgSO_4_, 7.5 mg/L FeSO_4_, 2 g/L yeast extract and 10 g/L glucose, pH 7.0. Glycine (2 g/L) was added to serve as the substrate for the C4 pathway. The media were supplemented with 100 μg/mL ampicillin (Amp), 50 μg/mL kanamycin (Kan) or 25 μg/mL chloramphenicol (Chl) accordingly. To induce expression of plasmid-borne genes, Isopropyl-β-D-thiogalactopyranoside (IPTG) was added to cultures which resulted in a final concentration of 0.1 mM. Considering cell growth and ALA stability, the pH was measured by a glass electrode and controlled at 6.5 ± 0.3 with 4 M NaOH. For RGMS, the modified CAYE medium (CAYEM) was used which contains 1.86 g/L citric acid, 9 g/L KH_2_SO_4_, 6 g/L (NH_4_)_2_HPO_4_, 0.6 mg/L MgSO_4_, 7.5 mg/L FeSO_4_, 2 g/L yeast extract and 4 g/L glucose. Considering cell growth and ALA stability, the pH was measured by a glass electrode and controlled at 6.0 ± 0.5 with 4 M NaOH.

Flask cultivations were carried out in 100 mL conical flasks supplied with 30 mL medium at 37 ℃ (or 30 ℃) with agitation of 200 rpm. A 1% (v/v) inoculum from an overnight culture was used. Cells were cultured in CAYE at 37 ℃ for 4 h until OD600 reach 0.7. Then IPTG with a final concentration of 0.1 mM was added for induction and induced fermentation lasted for 24 h. Batch fermentation was performed in 5 L fermenter containing 3.5 L CAYE medium. A 10% (v/v) inoculum from seed cultures was used. Seed cultures were obtained by batch fermentation, which was performed in 5 L fermenter containing 3.5 L CAYE medium and grown at 37 ℃ for 12 h, pH 6.9. To obtain seed cultures, a 1% (v/v) inoculum from an overnight culture was used. Glucose as a carbon source was added at initial with a concentration of 20 g/L. Fermentation was operated at 37 ℃ for 6 h and then IPTG with a final concentration of 0.1 mM was added for induction. After induction, the cultivation temperature was switched to 30 ℃. The whole fermentation was conducted at 30% dissolved oxygen (automatically adjusted with aeration and agitation rates), and pH 6.0 (automatically adjusted with H_2_SO_4_ and ammonium hydroxide). Solutions of glucose (700 g/L) and glycine (20 g/L) were fed into the fermenter to maintain the glucose concentration between 1 and 10 g/L, and to supplement glycine with the speed of 0.1–0.2 g/L/h.

### DNA isolation, manipulation, and PCR

MiniBEST Bacteria Genomic DNA Extraction Kit (Takara, Japan) was used for DNA isolation of *E. coli.* Cultures for chromosomal DNA isolation from *E. coli* were grown in LB. Oligonucleotide primers used in this work are listed in Table S2. Phusion® High-Fidelity PCR Master Mix (NEB, England) was used in the PCR reaction. PCR-amplified fragments were subcloned into specific vectors by In-Fusion® HD Cloning Kit (Takara, Japan). Recombinant plasmids were sequenced to confirm the correct construction.

### Construction of plasmids

Table S2 contains all primer pairs designed for gene cloning and plasmid construction. Plasmid pCas (Fig. S5A) and pTarget were prepared in our lab. Plasmid pTarget-gene was constructed by Inverse-PCR and T4-Ligation (T4 DNA Ligase, NEB, England) to replace the N20 fragment (Fig. S5B) with specific primers (Table S2). Plasmid pACYCD-gene and pACYCD-Donor were both constructed for preparing Donor DNA (Fig. S6) by In-Fusion® HD Cloning Kit (Takara, Japan). Donor DNA was cloned from pACYCD-Donor by Hi-Fi PCR (Phusion® High-Fidelity PCR Master Mix, NEB, England). The yellow fluorescent vector pUC57-sYFP was used to construct a basic reporter plasmid. Promoter of *hemL* (*PhemL*) was cloned from the genome of *E. coli* BW25113-T7 by Hi-Fi PCR (Fig. S7). The amplified 186 bp fragments, *PhemL*, were inserted into pUC57-sYFP to obtain pUC57-sYFP-pHemL by In-Fusion® HD Cloning Kit (Fig. S7). The coding sequences (CDS) of *sodB* and *katE* were cloned from genome of *E. coli* BW25113-T7 by Hi-Fi PCR and were inserted to pACYCD-hemA-RS respectively to obtain pACYCD-AS, pACYCD-AK or pACYCD-ASK. All plasmids used in this research will be listed in Table 3.

### Selection of integration site and design of homologous recombination

The function and detailed message of the *aceB* gene (and other genes) was verified in KEGG, BioCyc Database and NCBI. Sequence of the *aceB* gene (and other genes) in *E. coli* genome was confirmed in NCBI. As the recognition site for sgRNA, N20 site directs the Cas9 protein to enable site-specific induction of a DSB. The N20 site was found by BROAD international design tool, which is available at: http://www.broadinstitute.org/rnai/ public/analysis-tools/sgrna-design.

### Gene inactivation, insertion or replacement

The mutants *E. coli* A2 (*E. coli* BW25113-T7 Δ*aceB*, Δ*dppA*, Δ*hemF*, Δ*galR* int *ppc* with J23119, Δ*poxB* int *glk* with J23119), *E. coli* 108 (*E. coli* A2 Δ*PhemL* int J23018) and *E. coli* 111 (*E. coli* A2 Δ*PhemL* int J23111) were created using CRISPR/Cas9 mediated gene modification [52]. This method includes the following steps.

For transformation, the plasmid or linear DNA were electroporated into competent cells in the pre-chilled cuvette (0.1 cm) using Bio-Rad MicroPulser (1.8 kV, time constant > 5.0 ms). For selection, 25 μg/mL Chl or 50 μg/mL Kan were used alone or in combination. For induction of λ-Red proteins and lac operator, 1 mM arabinose and 1 mM IPTG were used. To prepare cells harboring pCas, cells cultured at 37 ℃ (OD600 = 0.45–0.55) were made competent, mixed with pCas (100 ng) and subjected to electroporation, after which the cells were recovered in SOC medium (1 mL) for 1 h at 30 ℃, plated onto the Kan plate, and cultured at 30 ℃ for 18–24 h. For CRISPR/Cas9-mediated homologous recombination, cells harboring pCas were cultured at 30 ℃ in medium containing Kan and Arabinose and made competent. After co-electroporation of Donor DNA (400 ng) with pTarget-Gene (100 ng), cells were recovered in SOC (1 mL) medium for 1 h at 30 ℃, plated onto Chl/Kan plate, and cultured at 30 ℃ for 18–24 h. For elimination of pTarget-Gene, cells harboring both pCas and pTarget were cultured at 30 ℃ in medium containing Kan and IPTG for 2 h. Cells were plated onto Kan plates and cultured at 30 ℃ for 18–24 h. For elimination of pCas, cells harboring pCas were cultured at 37 ℃ in the medium without any antibiotic for 12–16 h. Then the cells were plated onto non-antibiotic plates and cultured at 37 ℃ for 12–16 h. Schematic illustration of the CRISPR/Cas9 mediated gene modification was shown in Fig. S8.

### Analytical procedures

For analyzing ALA production, culture was centrifuged (12,000 × *g* for 2 min at 4 ℃). The supernatant was analyzed for extracellular ALA concentration. ALA concentration was analyzed using modified Ehrlich’s reagent [53]. Specifically, standard or sample (2 ml after diluted) was mixed with 1 ml 1.0 M sodium acetate (pH 4.6) in a cuvette, and 0.5 ml acetylacetone (2,4-pentanedione) was added to each cuvette. Then the mixtures were heated to 100 ℃ for 15 min. After cooling for 15 min, the reaction mixture (1 ml) and freshly prepared modified Ehrlich's reagent (1 ml) were mixed together. After 30 min, the absorbance at 554 nm was measured. Standard plot for ALA measurement is shown in Fig. S9.

For analyzing glucose, 1 mL of culture was centrifuged (12,000*g* for 2 min at 4 ℃) and the supernatant was then filtered through a 0.22 mm syringe filter for analysis. The HPLC system was equipped with a cation exchange column (HPX-87 H, BioRad Labs), and a differential refractive index (RI) detector (Shimadzu RID-10 A). A 0.5 mL/min mobile phase using 5 mM H_2_SO_4_ solution was applied to the column. The column was operated at 65 ℃.

Data for ALA production of were subjected to analysis of variance (ANOVA) by GraphPad Prism (version 7.00). Error bars indicate standard error of the mean (SEM). *P* values were calculated using Dunnett's multiple comparisons test (*P < 0.05, **P < 0.01, ***P < 0.001, ****P < 0.0001). The mean of each column was compared with the mean of a control column as indicated.

### Single-reporter RGMS

Fluorescent plasmid pUC57-sYFP-pHemL was introduced into *E. coli* BW25113-T7 by electroporation and the resulting transformants were designated *E. coli* BWT7-sYFP-L. Mutagenized CDS of *eamA* gene was cloned from pSC-LA-eamA by Diversify PCR Random Mutagenesis (Takara, Japan). Then those mutagenized fragments were inserted to replace the original *eamA* gene in pSC-LA-eamA by In-Fusion® HD Cloning Kit (Takara, Japan). These mutagenized plasmids were introduced into *E. coli* BWT7-sYFP-L by electroporation and mutagenized cells were spread on a 190-mm plate containing LB medium supplemented with agar (20 g/l), Chl (25 mg/ml) and Kan (50 mg/ml). Plates containing mutagenized cells were incubated at 30 ℃ overnight before colony picking. For single-reporter RGMS of fluorescent selection, colonies were randomly picked and cultured on CAYEM to measure fluorescence intensity. To measure fluorescence intensity, the fermented liquid was added to a 96 well plate after 24 h of induction. Then accurate fluorescent data was detected by Synergy™ HTX Multifunctional Microplate Detector (BioTek Instruments, America). The excitation light was set as 503 nm and the receiving light was set as 540 nm. Collected data was analyzed with the Gen5™ V2 Data Analysis Software (BioTek Instruments, Inc.). Approximately 6 ~ 10 mutants which showed strong fluorescence were selected to measure ALA production without replications. Then 2 ~ 3 mutants which have high titers of ALA production were isolated and remeasured with three replications. Finally, an optimal mutant was obtained through these steps. These steps can be one cycle of single-reported RGMS. The optimal mutant of *eamA* gene obtained in last cycle could be the template of next cycle till the ALA production reaches our expectation or mutagenesis reaches saturation. In this study, four cycles were conducted and each cycle was named as A, B, C and D cycles sequentially. Schematic illustration of RGMS process we designed in this study was shown in Fig. S2.

### Supplementary Information

Supplementary Material 1. Fig. S1. Growth characteristic of mutant *E. coli* strains in different medium. Cells were cultured in CA9YE (A) or LB (B) medium. Data are means of three replicates.

Supplementary Material 2. Fig. S2. Schematic illustration of modified single-reporter RGMS process in this study.

Supplementary Material 3. Fig. S3. Expression of sYFP under control of *PhemB* and J23108 promoter in different bacterial strains. The fluorescent signal was detected by Multimode Reader (read: 503, 540) and was normalized to the cell density (OD600).

Supplementary Material 4. Fig. S4. The reactions catalyzed or repressed by corresponding genes. The reaction directions shown in accordance with the physiological direction of the reaction. Blue arrows indicate decreased activity by expression of *galR*.

Supplementary Material 5. Fig. S5. Map of plasmids which were constructed for CRISPR. (A) Map of pCas, which harbored the temperature sensitive oriR101 with repA101ts, kanamycin resistance gene, the λ-Red operon encoding Gam, Bet, and Exo proteins under the control of arabinose-inducible promoter ParaB, *S. pyogenes*-derived cas9 driven by endogenous promoters and sgRNA guided to ori-p15a which is under the control of lac operator. (B) Map of pTarget-gene, which harbored Chloramphenicol resistance, ori-p15a and sgRNA guided to *E. coli* BW25113-T7 targeted gene.

Supplementary Material 6. Fig. S6. Construct of intermediate cloning vectors for preparing Donor DNA. (A) Fragment A cloned from BW25113-T7 was concatenated to pACYCD-Blank to assemble pACYCD-gene. (B) Reverse-PCR to constructed pACYCD-Donor. (C) The map of Donor DNA, which contains HRL and HRR.

Supplementary Material 7. Fig. S7. Construct of RGMS reporter vectors pUC57-sYFP-pHemL.

Supplementary Material 8. Fig. S8. Schematic illustration of DSB induction and homologous recombination. After preparing competent cells, the pTarget-gene and Donor DNA which harbored homology arms (HRR and HRL) that targeted a chromosomal locus spanning the middle of targeted gene and the DSB site were electroporated into cells.

Supplementary Material 9. Fig. S9. Standard plot for ALA measurement. x: the absorbance at 554 nm; y: ALA concentration of sample after diluted (mg/L).

Supplementary Material 10. Table. S1. The expression levels of constitutive promoter which is available in iGEM.

Supplementary Material 11. Table. S2. Primers for Plasmid constructions and Testing.

## Data Availability

The majority of data generated or analyzed during this study are included in this published article or in the supplementary information. Data not shown in this manuscript are available upon request from the corresponding author.

## Abbreviations

*E. coli*: *Escherichia coli*
ALA: 5-Aminolevulinic acid
ALAS: 5-Aminolevulinate synthase
GSA: Glutamate-1-semialdehyde
PBG: Porphobilinogen
CPG: Coproporphyrinogen
PPG: Protoporphyrinogen
Kan: Kanamycin
Amp: Ampicillin
Chl: Chloramphenicol
CRISPR: Clustered regularly interspaced short palindromic repeats
sgRNA: Small-guide RNA
HRL: Homologous left arm
HRR: Homologous right arm
ROS: Reactive oxygen species
IPTG: Isopropyl-β-D-thiogalactopyranoside
TCA: Tricarboxylic acid cycle
CDS: Coding sequences
SOD: Superoxide dismutase
CAT: Catalase
asRNAs: Antisense RNAs
